# Transition: The Patient in a No-Man´s Land

**DOI:** 10.1590/S1677-5538.IBJU.2025.0344

**Published:** 2025-05-20

**Authors:** Lisieux Eyer de Jesus

**Affiliations:** 1 Hospital Universitário Antônio Pedro Departamento de Cirurgia Pediátrica Niterói RJ Brasil Departamento de Cirurgia Pediátrica, Hospital Universitário Antônio Pedro, Niterói, RJ, Brasil;; 2 Hospital Federal dos Servidores do Estado Departamento, Cirurgia Pediátrica Rio de Janeiro RJ Brasil Departamento, Cirurgia Pediátrica, Hospital Federal dos Servidores do Estado, Rio de Janeiro, RJ, Brasil

**Keywords:** congenital [Subheading], Infant, Newborn, Diseases, Pediatrics

## Abstract

**Introduction:**

The need for transition from pediatric to adult health services developed from better survival rates of congenital diseases. Quality of life, Independence, and health preservation are to be considered. Perspectives on transition differ between patients, families, health professional and administrators.

**Methods:**

Non-systematic review and discussion of transition dilemas in a structured format.

**Results:**

Medical transition occurs during late adolescence/ early adulthood. Patients are expected to have increased longevity, need lifeterm follow-up and present varying degrees of complexity. Patients’ fundamental wishes may conflict with caregivers’ considerations. Technical decisions must be balanced, taking into account patient´s social, cognitive, motor and psychological conditions, and the needs of their family caregivers. Difficulties by pediatric healthcare professionals in mastering adult diseases, and challenges encountered by adult-specialized physicians in managing sequelae or ensuring continuity of care for pediatric conditions, are the rule. Patients afflicted by with chronic complex conditions may prove financially challenging. For families, transitioning from from paediatric care to adult-oriented medicine is destabilizing and distressing. The idealization of an unattainable normality is prevalent and detrimental, leading to the abandonment of therapeutic strategies deemed socially undesirable.

**Discussion:**

Transition care urgently requires planning by health administrators. Patients should be transferred to adult care only after clinical stabilization. Three models of transition have been suggested (direct transfer, protocol and age-driven; permanence under pediatric assistants control with eventual consultation with adult experts and gradual transition, permeated by a period of conjoint assistance). The last format is considered ideal.

## INTRODUCTION

The need for a transition mechanism for patients with congenital diseases or their sequelae (DCS) from pediatric care to adult specialist services is, in a sense, a recent development, and has quickly become a significant issue. Several general reference websites have been made available, all from high-income countries (e.g., http://www.gottransition.org, http://www.med.unc.edu/transition), as well as some guidelines, most of which are directed at populations with spina bífida (1).

The increase in survival rates—and, more importantly, the possibility of autonomous living with a full social life, including a payed job—has provided these patients with a perspective of adulthood, expectations of better quality of life and, at least to some extent, independence, depending on individual cases. Beyond preserving physical health, enabling the best possible quality of life on a case-by-case basis and encouraging independence and self-confidence are priorities (2).

### Transition

From a medical point of view, is the formal process that allows an adolescent or young adult (AYA) with a DCS to assume management of their health condition and to move from specialized pediatric care to sector-specific adult care. The goal is to provide assistance with appropriate perspectives and expertise tailored to mature and self-responsible individuals and to the health needs of the adult population. For healthcare managers and system infrastructure, this process enables optimal resource management, including equipment and logistics, the creation of new slots for high-complexity pediatric patients, planning for AYA DCS treatment, and monitoring long-term complex congenital diseases treatment outcomes.

### Transition is a process

Not merely an administrative age-based moment. The simple formal transfer of AYAs to adult services—accompanied by a break in the pediatric healthcare relationship—often proves ineffective, frequently resulting in informal returns to initial providers, therapeutic abandonment, refusal of acceptance by adult sectors, or loss to follow-up, with potentially serious health consequences (3). A recent paper reported that 2/3 of a cohort of North-American AYA with DCS reported difficulties to access medical care, and circa ¼ were not enrolled in adult clinics after 21 years of age (4).

Remaining under pediatric care without a proper transition is not without problems either. AYAs have clinical needs beyond the usual scope of pediatric specialists, including issues related to sexual and reproductive health, surveillance and treatment of adult and elderly diseases—often exacerbated or accelerated by their pediatric condition (e.g., tumor surveillance, cardiovascular and renal diseases, orthopedic issues, osteopenia). For example, children cured of pediatric tumors after chemotherapy and/or radiotherapy may have long-term sequelae, secondary tumor risks, high infertility risk, and, whenever gametes were preserved before treatment, a predicted need for reproductive specialist care.

An effective transition process requires:

1. Planning and assessing the patient/family's readiness for the change in healthcare sources.
2. Availability of mechanisms for control and monitoring after transfer to detect issues and prevent therapeutic abandonment—potentially affecting up to half of patients with chronic diseases (2).
3. Adaptation of receiving services to meet the logistical and practical needs of AYAs with DCS, involving multidisciplinary care, social support, financial assistance, and accessibility—fundamentally different from adult treatment models designed for isolated diseases in mature age.

In this paper we propose to discuss the challenges involved for transition from different perspectives:

## METHODS

Non-systematic review and discussion of transition dilemas in a structured format, regarding:

1. Relational Aspects Between Patients with Congenital Diseases, Their Families, and the Healthcare Teams Throughout Life Until Adulthood;
2. Technical Aspects Related to the Sequelae of Congenital Diseases and Their Treatments, Often Unfamiliar to Adult Medicine Experts;
3. Structural Aspects of Public and Private Healthcare Systems Concerning the Care of These Patients.

## RESULTS

Typically, medical transition occurs during late adolescence/ early adulthood (between 14 and 21 years of age). This population is expected to have increased longevity and necessitates long-term follow-up, often throughout their entire lives to manage current and potential health issues.

For the same underlying condition, different needs may arise. Individuals with congenital diseases exhibit varying degrees of complexity, diverse therapeutic outcomes, and frequent associations with comorbidities and syndromes. The results and needs can differ depending on the timing of diagnosis (antenatal versus early versus late), previous treatment strategies, modifications in the standard of care during the patient's growth, treatment choice for the specific patient conditions (including social and familiar issues), and the feasibility of therapeutic planning within the social context, availability of resources, and technological advancements.

As an example, an 18-year-old patient post-treatment for posterior urethral valves may be a clinically stable young adult requiring renal and cardiovascular monitoring; a young man facing fertility issues; a stable young individual dependent on intermittent clean catheterization; a renal transplant recipient; or a person on dialysis with severe hypertension. Also, the management of patients with urinary incontinence varies depending on access to costly hygiene supplies and the patient's motor capacity. In other words, the needs of individuals with Congenital Urological Conditions are highly individualized and variable, defying a single standardized guideline—"one size fits all", and this is true for life.

### Social and cognitive factors influence the timing of transition, which cannot be based solely on age.

The evolution of each adolescent through maturity differs, especially in the context of chronic illness. Chronological age may not suffice to classify patients with DCS as adults. Patients with significant physical dependence or severe cognitive limitations require tailored approaches and often have different future prospects compared to others. Some professionals suggest that certain adults with DCS and high dependency—particularly those with syndromic conditions involving cognitive and growth deficits—might need to remain under pediatric care, given their strict and permanent reliance on family caregivers and their inability to assume personal care responsibilities.

In the Brazilian context, social and logistical support for dependent patients within public health services remain very limited in practice, despite inclusive legislation. Similarly, most private health assistance institutions provide minimal ongoing support in terms of supplies, medications, and home care assistance. Consequently, the ongoing influence of close family members on therapeutic decisions persists long-term, sometimes involving ethically complex or conflicting choices, alongside the practical involvement of caregivers in daily needs.

### Considerations regarding the quality of life of family members and caregivers, including financial commitments, must be integrated into the transition process.

Patients’ fundamental wishes may conflict with their caregivers’ considerations. For instance, what should be done if parents of an adolescent with severe cognitive limitations request sterilization? Or when a teenage girl with spina bifida requests contraception, while her family refuses to acknowledge the possibility of an active sexual life? How can we balance the constant and lifetime need for support for children with DCS with the relational and financial needs of parents and siblings, aiming for fairness and justice?

Technical decisions must be carefully balanced, taking into account social, cognitive, and psychological conditions of the patients, their degree of dependence, and the needs of their family caregivers. Some decisions are particularly challenging, requiring a delicate balance between clinical needs, ethical considerations (such as ableism and social prejudices), the disproportionate and ongoing sacrifices of caregivers, and legal aspects. Practical circumstances must be considered realistically, including logistical and financial factors. Occasionally, the ideal outlined by technical guidelines may be unattainable, necessitating case-by-case decision-making. A common example among urological patients is the preferential choice of incontinent diversions for individuals with severe cognitive limitations, limited social expectations, or inability to perform self-care—particularly when family members need to work and lack adequate support structures.

Dysmorphia, social isolation, and unrealistic expectations are frequent among DCS patients. Suicidal ideation is not uncommon. Psychosexual concerns related to the penis following surgeries for hypospadias and epispadias are typical, as are distress regarding genital aesthetics and dyspareunia in female patients after genital reconstruction surgeries. Questions about fertility and the adoption of erroneous perspectives concerning the genetic transmission of diseases are also prevalent within this patient group. These issues typically emerge during adolescence and early adulthood, requiring integrated multidisciplinary care, including consultations with mental health and quality of life specialists, as well as social workers—services that are rarely available within pediatric or adult healthcare systems.

### Technical Aspects Related to the Sequelae of Congenital Diseases and Their Treatments, Often Unfamiliar to Adult Medicine Specialists:

The difficulties faced by pediatric healthcare professionals in mastering adult diseases, as well as the challenges encountered by adult-specialized physicians in managing sequelae or ensuring continuity of care for pediatric conditions, are remarcable. The increasing sophistication and expansion of medical knowledge have led professionals to focus predominantly on technical content within their usual domains or specific patient populations. This trend is evident even among generalists and emergency physicians specialized in adult medicine. The rarity and variability of many congenital diseases further complicate matters, as do the multidisciplinary aspects typical of patients with high-complexity conditions. Another significant issue is the scarcity of reliable guidelines for the treatment of patients with DCS (3).

In the current landscape of medical practice and training curricula, the education of physicians specialized in adult medicine regarding pediatric and congenital diseases remains limited to foundational knowledge. This can be particularly challenging for physicians which primarily or exclusively treat adults to care for patients with DCS, especially those with rare conditions.

A recent study revealed that only 5% of gynecologists in a U.S. healthcare facility had received formal training in pediatric and adolescent gynecology, and merely 7% of the institutions employing these professionals offered outpatient services for children and adolescents. Within this cohort, approximately one-third of physicians reported having performed a perineal examination on a patient with anorectal malformation, and only 12% and 5% had previously managed patients with appendicovesicostomy or appendicocolostomy, respectively. Moreover, 80% of these gynecologists expressed discomfort, and 82% considered themselves incompetent to treat adult patients with anorectal anomalies. Additionally, 22% explicitly indicated no interest in such patients, and 34% reported lacking the time to care for women with anorectal malformations. When responding to a basic questionnaire on anorectal and related anomalies and their implications in adult patients, approximately half of the professionals answered correctly only up to two out of seven questions (5). The knowledge and recognition of sequelae stemming from congenital diseases, as well as the possible adaptations and prognoses, are not easily accessible among adult medicine specialists, that often show limited interest in working with this population (6).

On the other side, pediatric experts do not master certain aspects of adult care, particularly concerning degenerative or aging-related diseases, reproduction and sexuality, as well as early detection and prevention of neoplasms.

As a consequence, lack of training regarding chronic illnesses and sequelae of congenital diseases care is a common complaint among both adult and pediatric physicians (2) and situate DCS patients in a "no-man´s land".

### The structure of healthcare and logistical considerations for the care of adult patients with congenital and developmental syndromes (DCS) requiring ongoing treatment.

DCS patients often exhibit some degree of dependency and are frequently outside the labor market, especially in low- and middle-income countries. Many have had limited education due to logistical challenges, multiple hospitalizations, or cognitive impairments. These adults have been managed throughout their lives within pediatric care frameworks that focus on family involvement and the direct protection of immature patient's incapable of fully consenting or managing their needs.

Often, families adopt a protective role that permanently infantilizes the patient, impeding their assumption of responsibilities and fostering expectations of lasting dependence or subordination. This situation may be accepted by the patient, which then behaves passively, refraining from direct participation in their healthcare responsibilities. Conversely, it can lead to complete rejection of parental and medical guardianship once the individual attains legal adulthood, often resulting in abandonment of therapeutic guidelines. In ideal circumstances patients and families develop a co-participatory approach, balancing patient's needs and limitations with the aspiration for maximum autonomy.

The transition to care tailored for autonomous adult patients presents immediate adaptive challenges, which has led to the recommendation of gradual transition algorithms as ideal solutions. For patients with DCS, the structure of outpatient medical services for adults—often fragmented among various specialists without a centralized "overall coordination" (which, in pediatric cases, is typically managed by the pediatrician)—complicates multidisciplinary care. Frequently, patients must travel to different locations on separate days, to see various professionals, involving logistical arrangements that are both complex and costly for patients and their caregivers. From the patient's perspective, the structure of care for adults often appears worse in quality than the structure they previously relied on for treatment.

The philosophy of care aimed at autonomous adults demands that patients assume individual responsibilities—an expectation that patients with DCS are generally unaccustomed to. The numerous healthcare professionals involved in their care often struggle to communicate effectively among themselves, especially when working in different institutions or on different schedules. Emergency visits tend to become common, even when inappropriate, as these patients are highly complex, often with conditions beyond the full technical expertise of adult emergency physicians, and frequently use multiple medications and present serious comorbidities. Urgent care typically involves symptomatic treatment and recommendations to consult their primary clinicians. Patients often find themselves caught between the limitations of emergency services and difficulties to get immediate access to their usual healthcare providers, showing another version of the "no man´s land" dilemma.

In general hospitals equipped with pediatric surgical services, contact between the pediatric team and adult specialists is straightforward. However, for patients "graduated" from exclusively pediatric hospitals to adult care services, this process can be more challenging, due to institutional transfers. Well-founded technical reports and direct thorough access to the patient's medical records help, but they do not fully replace direct communication among professionals—discussions that include opinions, planning, updates on previously impractical schedules, and assessments of the relational dynamics between the patient, their family, and healthcare providers. There are practical difficulties in conveying a comprehensive account of a complex, multifaceted clinical trajectory, which may involve multiple professionals and a vast amount of documentation accumulated over what is conventionally an 18-year lifespan (7).

Some issues faced by these patients have deeply personal implications—such as erectile dysfunction, infertility, or contraception in highly dependent individuals—necessitating a strong doctor-patient relationship, which is often lacking between DCS patients and their new clinician post-transition, and sometimes raising difficult ethical questions. Confidentiality can be particularly critical, especially for minors without full legal responsibility capacity or adults with cognitive limitations. There is often a conflict between unsupervised meetings between the patient and the attending physician—allowing greater privacy and more personal discussions initiated by the patient—and the legal right of guardians to be present during consultations, alongside healthcare professionals’ concerns about potential accusations of abuse or misconduct involving minors or unaccompanied adults.

Ethical and legal quandaries are commonplace: Is sterilization ethically justifiable in highly dependent young patients or those afflicted with severe, genetically transmissible diseases? Ought patients carrying genetic disorders or with possible genetic determination be referred for assisted reproductive technologies? When is advising patients at high risk against desired pregnancies defensible? Can requests for vaginal delivery be reasonably accommodated in women with complex pelvic reconstructions, given the currently available substandard evidence favoring caesarean sections? What is the true risk of malignancy in young patients undergoing augmentation cystoplasty?

From a cost perspective view, patients afflicted with chronic complex conditions may prove financially disadvantageous to institutions. They require lifelong, often highly complex, periodic care, frequently encompassing expensive and repetitive routine procedures (dialysis, post-transplant management, physiotherapy, social and psychological support). Regrettably, they may become "undesirable" patients, rejected by some institutions or practitioners.

### Families of adult patients bearing permanent sequelae of their condition.

For families, the transition of AYA from paediatric care to adult-oriented medicine is a destabilizing and distressing juncture. The prospect of readjusting to new treatment methodologies and new medical teams after eighteen years of frequent contact with paediatric specialists is, at the very least, unsettling and challenging. A constant relationship of profound trust and intimacy, painstakingly built over time, will be inevitably superseded, irrespective of the wishes of patients and families. The proposed approach entails the progressive independence of patients where feasible, which is emotionally challenging for many families of DCS patients.

The recognition of genuine, imagined, or exaggerated risks (depending on the case) is common. As previously mentioned, the families of these patients are often overprotective. Also, family ties frequently fracture during the course of these children's development, and the incidence of only-children is elevated, leading to exceptionally strong bonds between mothers (typically the primary caregivers in cases of marital dissolution) and DCS patients. Often, a highly solid mother-child dyad is a form of survival under these circumstances, for the patient, that really needs intensive care, and also to the carer, that dedicates him-herself to the child, in disregard of other personal needs and projects. However, such a powerful relashionship may impede the independence of DCS young adults. For the caregiver this kind of "empty nest syndrome" can be intensely acute and painful.

### Normality versus best possibility

The trials of adolescence, challenging for all children, are more arduous and protracted for DCS AYA, compounded by physical, social, and psychological issues. The idealization of an unattainable normality is prevalent and directly detrimental, frequently leading to the abandonment of therapeutic strategies deemed socially undesirable (intermittent clean catheterization in cases of micturition dysfunction or augmentation cystoplasty being a typical example) or medications perceived as having socially deleterious side effects (for instance, post-transplant medications causing acne) (8).

Risky behaviours are commonplace, including permissive sexual relationships with the risk of unplanned pregnancies in female patients, substance abuse, and the adoption of inappropriate diets. Amidst fantasies of absolute normalcy, the complete abandonment of medical treatments is common, with some patients ultimately only seeking episodic emergency care during crises.

Maturation to accept the necessity of consistent treatments in exchange for improved quality of life (as opposed to a "normal" life) and health security versus the fantasy of normality of being "just like everyone else" is difficult, often delayed, and associated with episodes of depression. A harmonious family relationship and the provision of comprehensive information regarding prognosis and expected limitations by the healthcare professional are beneficial. Patient´s education given to patients requires reinforcement and repetition, increasing in complexity alongside the child/AYA's cognitive maturation, including "difficult" information involving the admission of the incurability of the disease or sequelae and the expectation of lifelong treatment.

For a subset of patients, the level of dependence on caregivers/family turns to be greater than necessary due to insecurity, overprotective parenting, immaturity, diminished personal expectations, and social and logistical challenges. Often, the patient relinquishes the autonomy that is possible, however limited.

Accessibility for individuals with disabilities can be exceedingly challenging, especially in low- and middle-income countries, diminishing the potential for autonomy. Financial dependency and the inability to enter the workforce are related problems, sometimes attributable to insufficient formal education. For some, the theoretical expectation of autonomy predicted by technical criteria is not practically attainable, and the perspectives of the Family include the forever financial dependence of the DCS Family member.

### Sexuality in a no-man´s land.

Sexuality is a social taboo that becomes more problematic following genital reconstructions, for patients with continence problems, those afflicted with spinal cord neurological conditions accompanied by paraplegia, and those with cognitive deficits. The evolution of society toward recognizing the expectation of active sexual lives and typical adult affective relationships in this population remains insufficient, and these young individuals are erroneously treated as persons without such prospects or desires, or who do not elicit this type of attention. For patients, insecurity regarding specific problems, exacerbated by episodes of bullying and fear of rejection by potential partners, is the norm, leading to delayed affective and sexual initiation, submission to risky behaviours/partners and episodes of violence or long-term bachelorship.

As a consequence of neglecting the potential for sexual relationships in this population, reproductive counselling is frequently omitted. Unplanned pregnancies and/or diagnoses of sexually transmitted infections are not uncommon.

In males presenting spinal neurological conditions, anorectal anomalies, and sequelae of extensive pelvic surgery, erectile dysfunction is commonplace.

In women with spina bifida, the risk of recurrence of the condition in their offspring is higher than in the general population. Pregnancy planning necessitates the prescription of high doses of folic acid for several months prior to the cessation of contraceptive methods, a matter that must be explained with lucidity to patients from the moment they possess sufficient comprehension.

Many malformations entail specific obstetric risks, and patients require care from professionals cognizant of their previous medical history, ideally beginning from the period when a pregnancy is contemplated. In certain instances, pregnancy necessitates alterations to pre-existing, chronic, and established management protocols. An example is the need for indwelling catheterization via abdominal catheterizable channels due to increased uterine growth after the second trimester of gestation, or the cessation of medications with the potential to cause embryological anomalies or obstetric complications. Following complex reconstructive surgeries, the presence of a surgeon and/or urologist experienced in the patient's condition during caesarean deliveries is imperative.

## DISCUSSION

Arising from all of these considerations, teams dedicated to the paediatric care of patients with chronic complex conditions have been seeking algorithms for the transfer of these young individuals to specialized adult care. This need urgently requires assumption by health administrators as well (2).

An initial premise is the complete stabilization of the patient prior to transfer. AYAs should be transferred in a clinical situation that presupposes the utilization of therapeutic strategies for chronic use, already accepted and assimilated, and the absence of unresolved acute problems.

The debate regarding transition mechanisms and the considerable variability among cases suggests that a single, rigid transition mechanism is untenable. Rigid healthcare management control mechanisms, including computerized guidelines that "do not accept" patient enrolment outside of the planned age range in paediatric care, are destined to perpetrate injustices that may ultimately result in loss of follow-up and clinical deterioration of patients, who end up as outcasts of the system.

Regrettably, this type of problem has been exceedingly frequent since the adoption of computerized classification systems based on artificial intelligence, modulated by non-specialized professionals possessed of limited decisional autonomy.

The models proposed for the transfer of patients with chronic complex conditions are (Figure-1):

### Direct, Protocol-Driven Transfer at a Single Point in Time:

Direct referral to specialized adult treatment services, severing the connection with paediatric care, mediated exclusively by the transfer of clinical data (detailed reports and/or copies of medical records). This is the standard procedure in most Brazilian maternal-child healthcare services not located within general hospitals, with the age range varying between 14 and 18 years. This model is the most frequently used, owing to its "automatism" and because it has, until recently, been presented as the sole alternative (with the exception of the "informal" continuation of patients under paediatric care after adulthood). The model of compulsory automatic transfer for treatment in services directed towards adults has been most strongly associated with loss of follow-up, clinical deterioration, and episodic care in emergency departments. Another issue is the non-acceptance of the transfer by the patients, who spontaneously return to the paediatric service and complain about what they judge the insuficiente technical knowledge of health adult-specialized providers about their specific condition. The perpetuation of previous treatments without medical supervision by patients is another form of reaction.

Continuation under Paediatric Treatment with Occasional Consultation in Adult Medicine:

Maintenance of patients under the care of paediatric professionals specialized in their underlying diseases, irrespective of age range, contingent upon the modulation of these professionals regarding the need for treatment of conditions typical of adults or specific circumstances (infertility treatment, obstetric treatment, contraception, for example), in a form of "shared management." This is a common format in general hospitals that houses paediatric and paediatric surgery services, and a frequent practice, especially among older professionals. Often, it is the patients who request the continuation or return to the care of the professionals with whom they have had a relationship since childhood.

### Gradual Transfer to Adult Medicine with an Intermediate Period of Overlapping Care:

Gradual transition from paediatric services to adult medicine, with an intermediate period of joint outpatient treatment by the paediatric professional and the adult specialist. This format is the most recently proposed and is considered ideal, as it fosters an initial familiarity of patients with their future attending physicians in a gradual manner, and also the gradual transfer of knowledge and information between professionals. However, it requires the confluence of medical specialists in paediatric medicine and adult medicine in the same physical environment for a prolonged period, which may present logistical difficulties, including with regard to the time availability of both professionals.

Other issues involve the inadequate adoption of approaches "approximating" adult disease management to treat conditions specific to the paediatric age range by adult specialists, and the "competition" of patients graduating from paediatric services for appointment slots that prioritize "conventional" adult patients and cases of acute urgency, particularly those with malignant neoplasms.

The survival of patients with neurological and behavioural disorders into adulthood, especially with regard to the alarming rates of autism spectrum disorder (ASD) in isolation or in association with other congenital conditions, and patients with cerebral palsy, presents a new challenge in the near future, which has been little studied and not even considered by most of the health administrators.
